# Oxygen-supplied electrotherapy for enhanced photodynamic synergistic therapy overcomes hypoxia tumor microenvironment

**DOI:** 10.1515/nanoph-2022-0417

**Published:** 2022-10-12

**Authors:** Chaozhou Li, Hui Tan, Ruitao Lu, Sainan Qin, Xiangying Meng, Han Zhang, Zhongjian Xie

**Affiliations:** International Collaborative Laboratory of 2D Materials for Optoelectronics Science and Technology of Ministry of Education, Institute of Microscale Optoelectronics, Shenzhen University, Shenzhen, 518060, P. R. China; Respiratory Department, Shenzhen Children’s Hospital, Shenzhen, 518038, China; Shenzhen International Institute for Biomedical Research, Shenzhen, 518116, Guangdong, China

**Keywords:** black phosphorus, electrotherapy, photodynamic therapy, ROS

## Abstract

Photodynamic therapy (PDT) has lately been identified as a promising anticancer method and gained tremendous interest due to its controllability, non-invasive nature, and negligible side effects. Nevertheless, the development of PDT is hampered by two factors. One is the insufficient tissue penetration of phototherapy laser, resulting in restricted treatment sites. Another one is the substantial dependence of reactive oxygen species (ROS) formation on oxygen concentration. Therefore, a strategy to promote ROS generation by overcoming the hypoxia microenvironment is critical to cancer therapy. Electrolysis of water is known to be a rapid and relatively secure method for producing oxygen. Thus, in this study, electrotherapy was introduced to alleviate the tumor hypoxia by producing oxygen *in situ*, hence boosting the PDT efficacy, namely E-PDT. Black phosphorus (BP) based nanomaterials were selected as clearable photosensitizers with outstanding PDT performance. Experiments conducted both *in vitro* and *in vivo* indicated that E-PDT performed superior therapeutic effects with the *in situ* generation of oxygen by electrotherapy compared with other groups. This work suggests a promising strategy for phototherapeutic anticancer efficiency enhancement.

## 1 Introduction

In clinical practice, the superficial tumor has been treated with photodynamic therapy (PDT) over the years according to its numerous advantages, including low adverse effects, non-invasiveness, minimal drug resistance, and light-controlled selectivity [1–3]. The photosensitizer (PS), light sources with the proper wavelengths, and dissolved oxygen in the cells are the three non-toxic components that formed the molecular process of PDT [4]. The electron transition from the ground state (S_0_) to the singlet excited state (S_1_ S_2_, S_3_, etc.) occurs when the PS is illuminated by adequate light [5]. Generally, the PS is divided into two categories based on the produced reactive oxygen species. Via electron transfer mechanism, the T_1_-stated PS engages with H_2_O or O_2_ to form reactive oxygen species (ROS), such as superoxide radical (O_2_^−^) and hydroxyl radical (·OH). This reaction is usually known as ‘type I PDT’ [6]. In contrast, a process known as ‘type II PDT’ is an energy transfer reaction that the hazardous singlet oxygen (^1^O_2_) is generated when the T_1_-stated PS potentially sensitizes O_2_^5^. Typically, a ROS molecule was produced from a PS molecule in type I PDT [6]. In comparison, ^1^O_2_ could be created continuously by a PS molecule in the presence of sufficient O_2_ and light in type II PDT [7]. Consequently, an excellent therapeutic effect could be obtained with only small doses of PS. As a result, ROS generated by PSs through the type II PDT mechanism has become quite popular in clinical applications. As mentioned above, oxygen is the substrate to produce toxic reactive oxygen species in PDT. Adequate oxygen supply is the prerequisite to ensuring sufficient effect of PDT. However, abnormal growth of tumor blood vessels and the malignant growth of cancer cells prevent enough oxygen from reaching the tumor tissues [8]. The efficacy of oxygen-dependent PDT would be much reduced due to the hypoxic tumor microenvironment (TME). Besides, the hypoxic TME could promote tumor growth and metastasis [9]. Therefore, it is essential to alleviate the hypoxia problem in tumor treatments.

Electrotherapy or electrical stimulation (ES) is the introduction of a low-level electric current into the human body to stimulate and activate muscles or nerves [10, 11]. It can treat orthopedic injuries and muscle-related diseases [12, 13], but its application in treating major diseases such as cancer is still an unknown blank. Guohua et al. innovatively combined the traditional Chinese acupuncture iron needles (electrodes) and *in vivo* electrochemistry to develop a cancer treatment method based on the selective electrochemical production of hydrogen in the tumor area. This method can rapidly generate hydrogen in the tumor area by electrolyzing H_2_O [14]. Because the theoretical voltage required for electrolysis of water is only 1.23 V, electrical treatment provides a precise and direct approach to creating oxygen [15]. Oxygen will generate fast at the anode when a direct current (DC) electrical power source is applied. Additionally, electrotherapy is a safe method of oxygen generation because there was no additional drug intake that might cause side effects when synergistically working with other therapies. To some extent, the hypoxia problem of the TME might be alleviated with electrotherapy. Wang’s group demonstrated that BP nanosheets are very efficient photosensitive catalysts for forming singlet oxygen with a quantum yield of around 0.91 [16], making them promising candidates for photodynamic treatment [17]. Besides, BP displays excellent biocompatibility and low toxicity because it could be degraded into phosphate and phosphorus products in the final [18, 19] has attracted widespread attention as PS.

**Figure 1:**
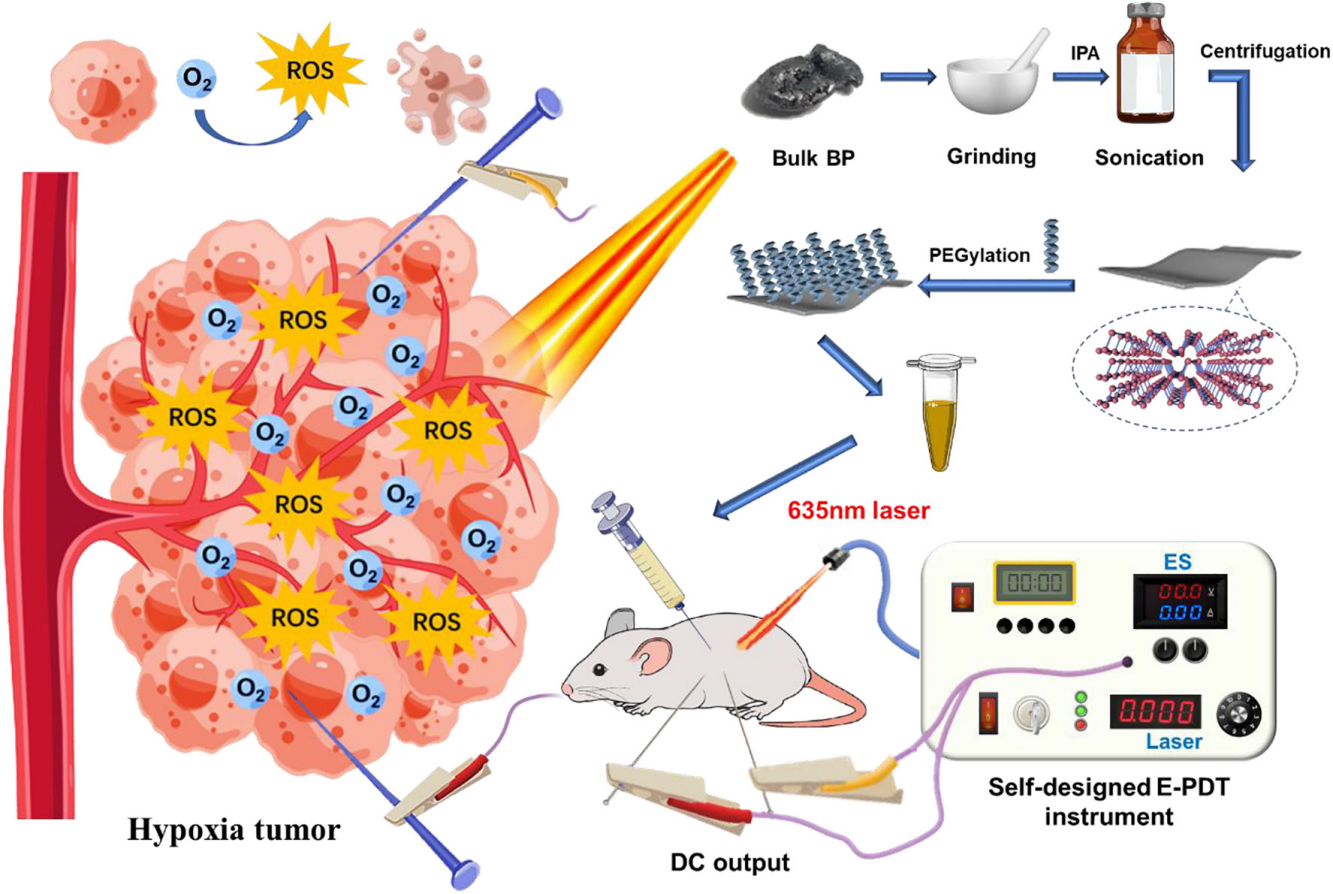
The schematic of sophisticated BPNSs for synergistic antitumor therapy.

To address the hypoxic tumor issues, modified BP nanosheets were applied to photodynamic/electrical synergistic cancer therapy (Figure 1). In this therapy system, mPEG-NH_2_ was decorated on the surface of BPNSs to improve photostability under irradiation of 635 nm laser and biocompatibility. Meanwhile, electrotherapy played as an oxygen donor role that relieved hypoxia for enhanced BP-mediated PDT. An E-PDT instrument was designed and conveniently utilized in both *in vitro* and *in vivo* studies. Herein, we proposed a novel tumor therapeutic that overcomes the fundamental limitations of PDT and exhibits remarkable tumor inhibition effects. This innovative E-PDT therapy offers a new possibility for the future treatment of hypoxic tumors.

## 2 Materials and methods

### 2.1 Instruments and materials

Semiconductor diode lasers, LSR635NL (Lasever, Inc.), were used as the excitation source for the 635 nm laser. The emission power was measured by a laser power meter (LM-3B20). A thermal imaging camera (FLIR E-75) was used to measure temperature change during the irradiation. The multimodal therapeutic was carried out by using the self-developed E-PDT instrument. The morphology characterizations of BP nanosheets were obtained on a transmission electron microscope (TEM) (FEI Talos F200S) and an atomic force microscope (AFM) (Bruker Icon). The absorption spectra were measured on a UV–Vis spectrophotometer (UV-2601, Beijing Beifen-Ruili Analytical Instrument). The size of nanosheets was detected by a NanoPlus-3 DLS instrument. Raman analysis was performed on a HORIBA Scientific LabRAM HR Evolution Raman micro-spectrometer with 514 nm excitation. On a K-Alpha device from Thermo Scientific, X-ray photoelectron spectroscopy (XPS) measurements were performed. An optical granulometer was used to measure the zeta potential (Zetasizer Nano ZS90, Malvern Instruments Ltd.).

Bulk BP was purchased from Nanjing Muke Tec Co., Ltd. mPEG-NH_2_ (1000Da) was provided by Shanghai Yayi Technology Co., Ltd. Phosphate buffered saline (PBS) (pH 7.4), Roswell Park Memorial Institute-1640 (RPMI-1640), Fetal Bovine Serum (FBS), trypsin-EDTA, and penicillin/streptomycin were purchased from Gibco Life Technologies. Dimethyl sulfoxide (DMSO) was gained from Sigma–Aldrich. A549 was gained from ATCC. CellTiter-Glo luminescent kit was brought from Promega Co., Ltd. Dead Cell Apoptosis Kit (Annexin V-FITC) and Calcein-AM/propidium iodide (PI) kit were obtained from Shanghai Beyotime Biotechnology Co., Ltd. DCFDA Cellular ROS Assay kit, all the antibodies for western blot analysis were purchased from Abcam. All the rest chemicals were purchased from Aladdin Reagents unless mentioned above. All the water employed in the experiment was ultrapure water at 25 °C and 18.25 MΩ cm.

### 2.2 Synthesis of BPNSs and BP-PEG platforms

The BP nanosheets (BPNS) were produced through the sonication liquid exfoliation of bulk BP [20]. Briefly, bulk BP was ground into nanoflakes and dispersed in IPA (1 mg/mL). The mixture was sonicated with an ultrasonic cell disruption apparatus in an ice bath for 8 h, and then the BP dispersion was further exfoliated in bath sonication for 12 h at 10 °C. Afterward, the obtained mixture was centrifuged (7000 rpm, 20 min) to eliminate the oversized BP. The suspension containing the expected size of BPNSs was centrifuged again (13,000 rpm, 30 min) and precipitates were rinsed with deionized water before being freeze-dried under vacuum. The fabricated BPNSs were stored in dark at 4 °C to avoid degradation. To modify the surface of BPNSs, mPEG-NH_2_ was added into BPNSs suspension at a mass ratio of 1:5. The mixture was stirred for 4 h to achieve well contact between these two compounds. The combination was then centrifuged at 10,000 rpm (4 °C) for 15 min twice to clean the excess mPEG-NH_2_. Finally, precipitates were freeze-dried after washing two times to obtain PEGylated BP nanosheets (BP-PEG).

### 2.3 Photodynamic performance of BP-based nanomaterials

The photodynamic effect analysis of BP-based nanomaterials was carried out in six groups. Electrotherapy was applied at before or after laser illumination to investigate the oxygen influence on the PDT further. Pure water was used as the negative control group. DPBF (50 μL, 10^−4^ mol/L) was added into BP-PEG in the dark. For the laser group, the samples were irradiated by laser (635 nm, 1 W/cm^2^). For electrical stimulation, a 2 V direct current was applied to the samples. All the samples were loaded in a 2 mL cuvette and measured by UV–vis at different times.

### 2.4 In vitro analysis

Human lung cancer cells (A549) were brought from Procell Life Sci-ence & Technology Co., Ltd, cultured in RPMI1640 medium containing 10% FBS and 1% penicillin–streptomycin, and incubated at 37 °C in a humidified atmosphere of 5% CO_2_.

#### 2.4.1 Cytotoxicity assay

The bioavailability of BP-based nanoplatform was carried out in A549 cells through luminescent assay. The A549 cells were seeded in the white opaque-walled 96-well plates (2 × 10^4^ cells/well) with culture medium overnight. After that, medium was replaced with 100 uL medium containing different concentrations of BP-PEG (5, 10, 25, 50 and 100 μg/mL). Followed by 24 h incubation at 37 °C, the cell was equilibrated at room temperature before adding CellTiter-Glo reagent. The luminescent signal, recorded through a luminometer, is positively correlated with cell viabilities.

#### 2.4.2 Cell live/dead analysis

In general, the phototherapeutic effect of BP-based platform was evaluated through Calcein AM/PI assay and quantified with the luminescent assay. The A549 cells were seeded into 12-well plates (2 × 10^5^ cells/well) overnight with complete medium. Then the cells were cultured with the medium containing BP-PEG for 4 h. After the cells were rinsed using PBS, laser irradiation treatment (635 nm, 0.5 W/cm^2^, 5 min) and electric treatment (2 V, 2 min) were selectively implemented in different cell groups. After treatment, cells were cultured at 37 °C for another 12 h. Before the examination using an inverted fluorescence microscope, Calcein-AM and PI were added to stain the cells for 30 min. The ratio of live/dead cells could reflect the phototherapeutic effect. For quantitative analysis, the cells were treated with the same conditions, and then the luminescent assay detailed above was conducted.

#### 2.4.3 ROS fluorescence imaging and flow cytometry analysis

For the ROS level measurement, the DCFDA kit was utilized to ascertain intracellular ROS production of A549 cells. The A549 cells were cultured for 24 h in a complete medium in 6-well plates and then replaced with BP-containing medium. After BP was taken by the cell, the irradiation treatment with/without electrotherapy was applied to different groups. The cell was incubated with DCFH-DA for 30 min and rinsed with buffer two times. The fluorescent images of cells were recorded by an inverted fluorescence microscope. The amount of generated ROS was also measured using BD FACSCalibur flow cytometry.

#### 2.4.4 Apoptosis assay

Seven groups of cells were seeded into the 6-well plates and then treated with different conditions. After laser illumination and electrical stimulation, the cells were incubated for another 12 h. Finally, cells were collected and resuspended for 15 min in a binding buffer containing propidium iodide (PI, 5 μL) and annexin-V FITC (5 μL) in the dark. Then the fluorescence intensity was examined by a flow cytometer. In addition, the western blot analysis of PRAP and caspase-3 was undergone as these two indicators are highly correlated with the cell apoptosis level [21, 22].

### 2.5 Animal and tumor model

Animal modeling was performed based on the Balb/c nude mice (6 weeks old, female). On the right flank of each mouse, PBS containing 1 × 10^6^ A549 cells (100 μL) was subcutaneously injected to create the tumor model. Tumor volume was calculated using the following formular [23]: Tumor volume (mm^3^) = 1/2 × length × width × width. When the tumors grew to approximately 100 mm^3^, mice were utilized for antitumor therapy. Seven groups of tumor-bearing mice were randomly assigned (*n* = 5) for different treatments: (1) PBS; (2) only ES; (3) only BP-PEG; (4) BP-PEG with laser; (5) BP-PEG with laser and then ES; (6) BP-PEG with ES and then laser; (7) BP-PEG with ES and laser simultaneously.

#### 2.5.1 BP-based nanomaterials *in vivo* anticancer performance

For group 1, the mice were intravenously injected PBS (50 μL). For group 2, the mice were only treated with electrotherapy (2 V, 2 min). For group 3, the mice obtained an intravenous injection of BP-PEG (50 μL). For group 4–7, the mice were given an intravenous injection of BP-PEG. After 1 h injection, the mice of those groups were applied for further therapy. For laser irradiation, the mice were illuminated by 635 nm laser (0.5 W/cm^2^, 10 min), and the temperature variations around tumor area were recorded using an infrared camera. The electrotherapy parameter was the same as group 2. The treatments were conducted every other day for the first five days. Afterward, the mice were photographed and assessment of the tumor volume using a caliper was undergone every other day. Additionally, the mice weights were noted. The survival rate of every group in this study was recorded.

#### 2.5.2 Histopathological and immunohistochemical analysis

After 21 days, all the mice were examined before execution. All the tumor tissues were dissected and photographed before being fixed in 4% paraformaldehyde. The main organs such as heart, liver, spleen, lung, and kidney were harvested and stored in 4% paraformaldehyde. Finally, both main organs and tumor tissues were sectioned and stained with hematoxylin and eosin (H & E) for pathological analysis. In addition, the tumor slices were analyzed utilizing TUNEL immunohistochemical staining assays.

## 3 Results and discussions

### 3.1 Characterization of BPNSs and BP-PEG

#### 3.1.1 Morphology of BP-based nanomaterials

**Figure 2:**
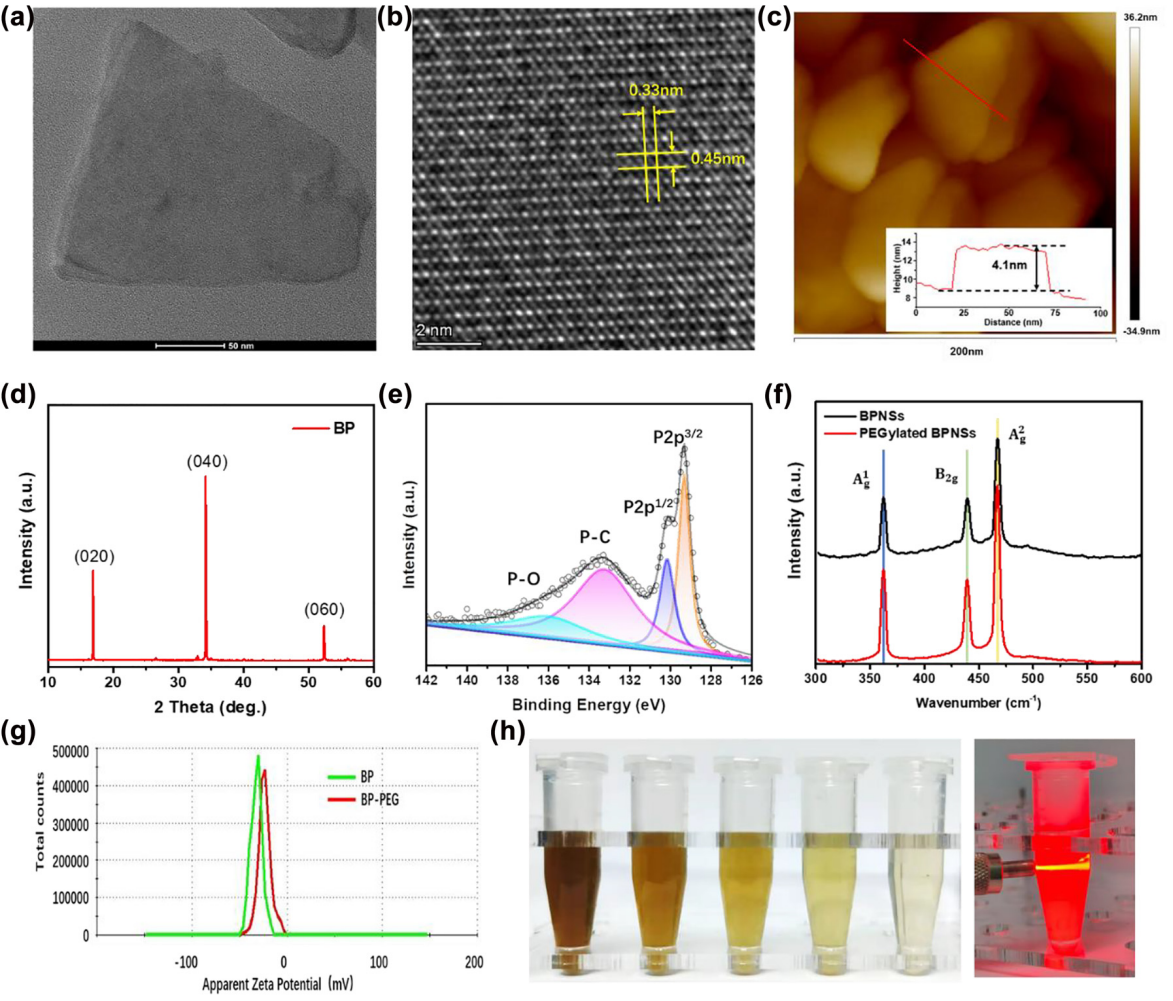
BP characterizations (a), (b) TEM images (c) AFM height analysis, (d) XRD spectra, (e) high-resolution XPS spectra of P2p, (f) Raman spectra of BP and BP-PEG, (g) zeta potential analysis of before and after PEG covered BP, (h) photographs of BP-PEG solutions with various concentration, BP dispersion with laser irradiation.

The morphology of BP-based nanomaterials was characterized using TEM and AFM. TEM image shown in Figure 2a discovered that the lateral dimensions of BPNSs were approximately 100–150 nm. The BPNSs diameter distribution before and after PEGylated was analyzed by DLS in the aqueous system as well (Figures S1 and S2). The DLS results revealed that the average particle diameter of nanomaterials increased because of the long PEG chain decoration on the BPNSs surface in water. Besides, due to the larger particle size after PEGylated, BP-PEG was less likely to accumulate in the kidney *in vivo*, lowering its toxicity. The few-layer BP’s crystallinity was examined using high-resolution TEM (HR-TEM) (Figure 2b). Lattice fringes were clearly shown on the BP atomic layer and consistent with the well-known BP lattice characteristics. The typical lattice fringes of BP indicate the BP nanosheets generated by exfoliation in IPA maintained their original crystallinity. According to AFM analysis, as shown in Figure 2c, the thickness was about 3.4 nm, which proved that few-layer of BPNSs were successfully produced. The (020), (040), and (060) planes shown in the XRD pattern of BPNSs (Figure 2d) demonstrated the excellent crystallinity of BPNSs same as as-synthesized BP. XPS was applied to detect the chemical composition of the BPNSs (Figure 2e). For the P2p spectra of BPNSs, the 2p^3/2^ and 2p^1/2^ exhibit at 129.8 and 130.7 eV, respectively, which are the representative binding energy of BP. In addition, the oxidized phosphorus (PO_x_) displayed at 135.9 eV, suggesting BPNSs were well protected in IPA. The P–C bond appeared at 134.2 eV indicating the strong connection between BP and PEG. Both bare BP and PEGylated BP were analyzed using the Raman spectrum (Figure 2f). The identical Raman signals of both samples exhibited peaks at 361.0 cm^−1^, 438.0 cm^−1,^ and 465.3 cm^−1,^ respectively. The Raman spectra suggested that no structural transformations happened during the BP nanomaterials preparation. Compared to bare BP, all three modes of PEGylated BP exhibited slightly red shift because of the adsorption after the PEG coating, which inhibited the phosphorus atoms’ oscillation and reduced the associated Raman scattering energy. In Figure 2g, the zeta potential of BP-PEG was less negative than BP, which also confirmed the surface of BPNSs was modified with mPEG-NH_2_. Various concentrations from 5 to 100 ug/mL of BP-PEG were presented in Figure 2h. These samples presented excellent dispersibility, and typical Tyndall effects were shown when irradiated with 635 nm laser.

#### 3.1.2 Photodynamic efficiency and stability of BPNSs

**Figure 3:**
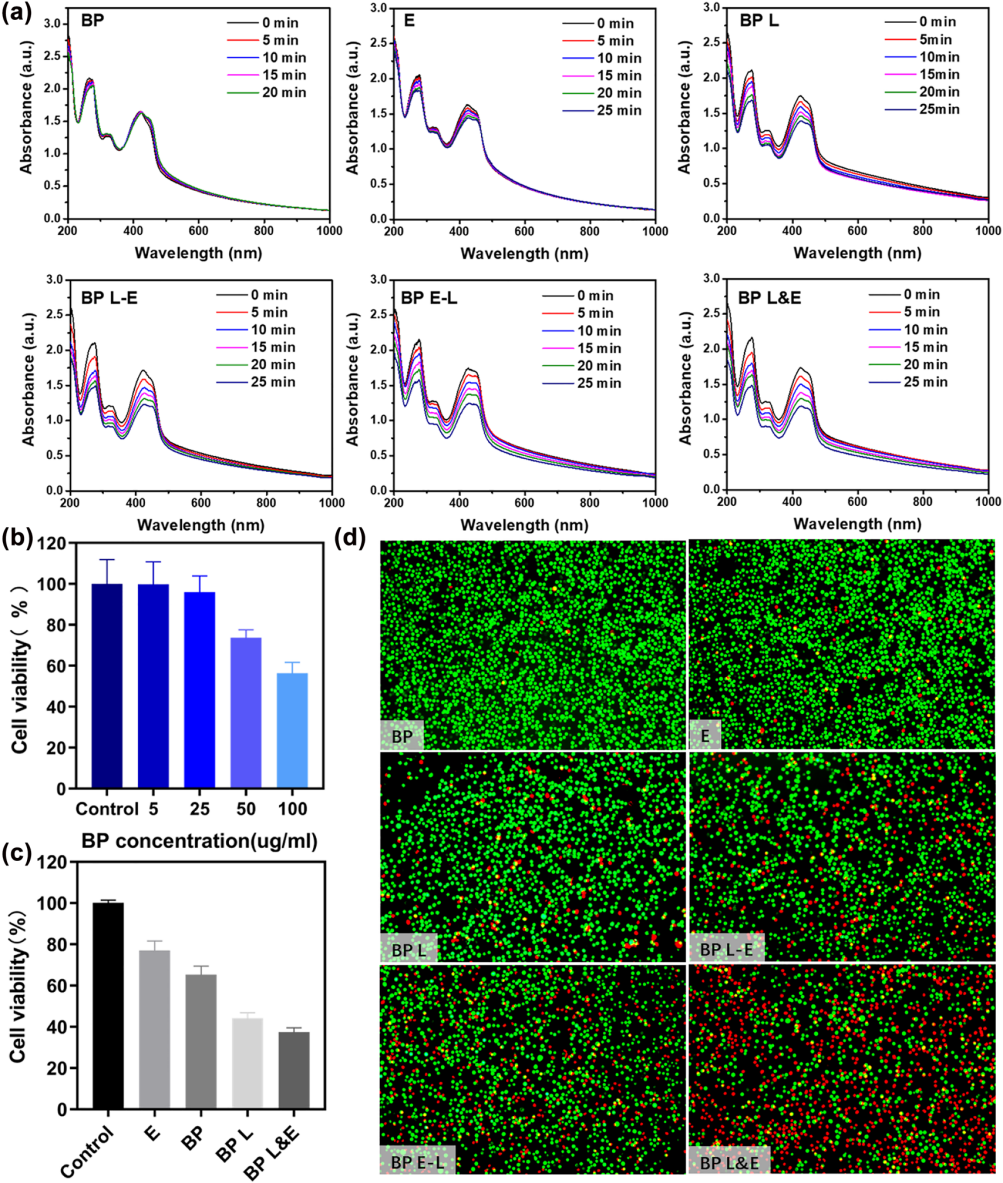
PDT effect and cell viability analysis of BP-based nanomaterials. (a) ROS detection, time-dependent absorption spectra of the DPBF with different conditions, (b) cell viability of A549 cells 24 h after incubated various BP concentrations, (c) cell viability of A549 cells applied with different treatments (d) fluorescence images of A549 cells stained with Calcein-AM (live cells, green fluorescence) and propidium iodide (dead cells, red fluorescence).

For the photodynamic properties of BP-based nanomaterials evaluation, the ROS generation amounts were monitored using DPBF via UV–VIS (Figure 3a). The characteristic absorption peak of DPBF at 420 nm decreased according to the irreversible reaction between ROS and DPBF. When DPBF solution was treated with BP or electrotherapy alone, the absorption peak remained essentially constant. The absorption peak of DPBF decreased with irradiation times. The characteristic peak fell dramatically after electrotherapy was applied. Compared with the BP L-E and BP E-L groups, the peak value decreased more rapidly when two therapies were conducted at the same time.

### 3.2 In vitro assessments using A549 cells

To assess whether the BP-based nanomaterials could be safely utilized for biomedical applications, the potential toxicities in A549 cells, at various concentrations were demonstrated in Figure 3b. It validated that the BP-based nanomaterials showed no significant cytotoxicity the cell viability was maintained 80% until the concentration achieved 50 μg/mL. Only at far above dose concentration (100 μg/mL) the cell viability dropped to 60% which confirms the excellent biocompatibility of the PEGylated BP. To further investigate the PDT and PTT performance of prepared nanomaterials, the cell live/dead analysis was carried out with A549. The statistics results of luminescent assay displayed in Figure 3c, only 37% of the cell still alive after phototherapy and electrotherapy. To reveal cell status post-treatment, Calcein-AM/PI analysis was applied (Figure 3d). It was shown that electrical stimulation cell impact was minor, so as in the BP group. Cell death gradually appeared after laser irradiation. For three groups that received both phototherapy and electrotherapy, the group in which two treatments were applied simultaneously presented powerful cell-killing effects. The difference in live/dead cell percentage between the other two groups could be negligible. Therefore, the introduced electrotherapy indeed promoted the antitumor effect. It was still unknown about the antitumor effect was attributed to PTT or PDT. The photothermal effect of BP under 635 nm laser irradiation was approved shown in Figure S4.

**Figure 4:**
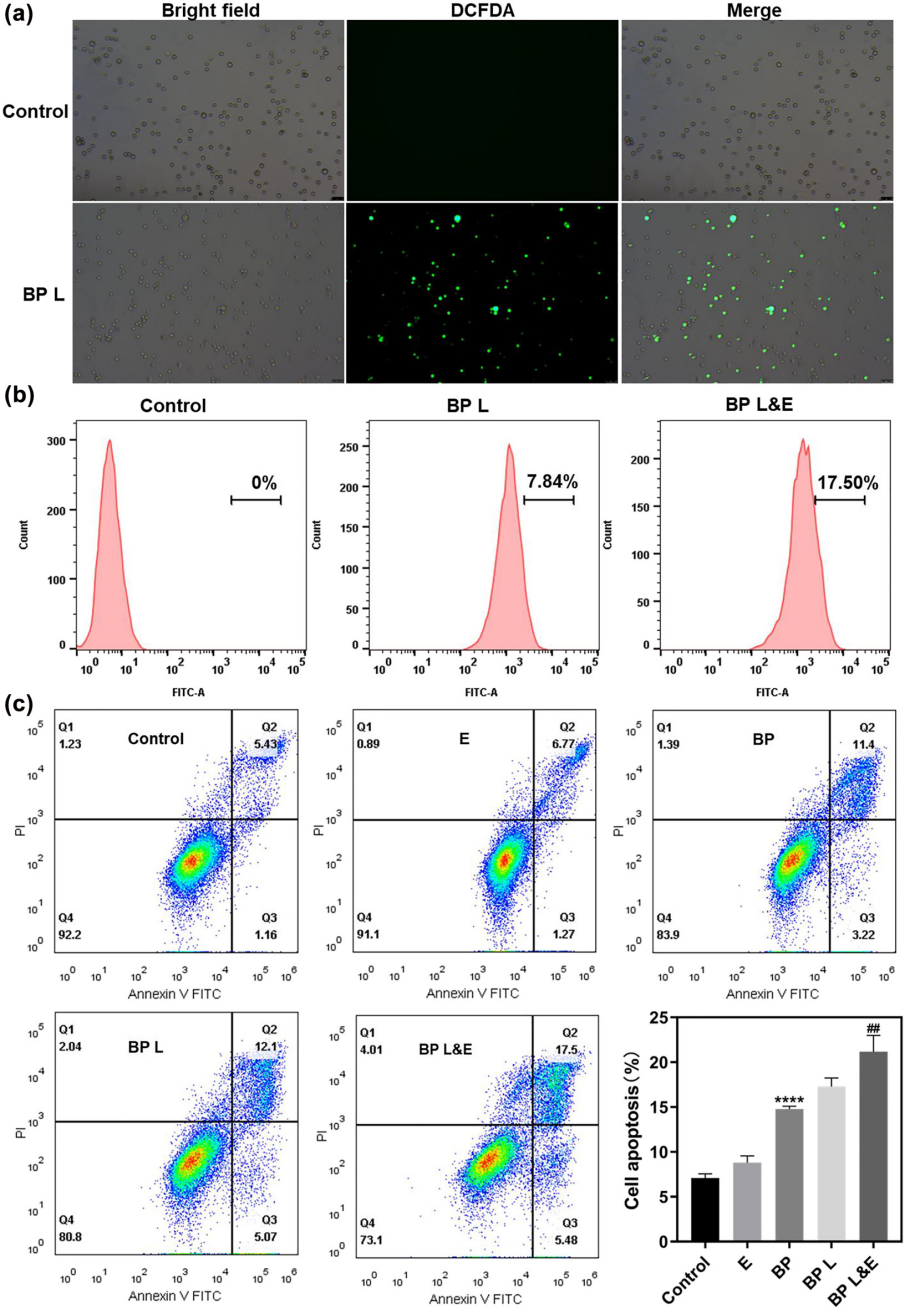
ROS detection and cell apoptotic analysis of BP-PEG *in vivo*. (a) DCFDA analysis of ROS after laser induced (b) flow cytometric analysis of ROS generation in A549 cells after indicated treatment in the culture medium without glucose. (c) Representative annexin V-FITC/PI scatter plots of A549 cells after 24 h of treatment (the bottom right quadrant displays early apoptotic cells, while the upper right quadrant displays late apoptotic cells).

Thus, the cellular ROS assay was implemented to detect whether the oxygen generated by electrotherapy promotes the photodynamic effect of BP-basednanomaterials. The preliminary ROS analysis was conducted using a DCFDA probe to label the cellular ROS of A549 because the DCFDA molecules could be converted to DCF by ROS, resulting in intense green fluorescence that serves as an indication of ROS production. The fluorescent images listed in Figure 4a indicated that green fluorescence was hardly observed in the control group. In contrast, a large amount of ROS was generated with laser as emitted strong fluorescence signals. Subsequently, the ROS generation level of different groups was quantified using flow cytometric analysis. As shown in Figure 4b, there is no ROS signal was detected in the control group. For BP L&E group, the signal reached 17.50% which was more than double the BP L group’s value (7.84%). It is approved that the electrotherapy improved the ROS generation as we expected. It confirmed that the oxygen generated during electrotherapy promoted ROS production. As the generated oxygen would be released from the medium with time, this oxygen source may not be interrupted with ROS generation when two therapy applied separately.

The necrotic and apoptotic assay of A549 cells induced by 635 nm irradiation was quantified utilizing flow cytometry. Positive labeling for Annexin V-FITC demonstrated early apoptosis, which was characterized by plasma membrane reconfiguration. Whereas cells were positive labeling Annexin V-FITC and PI owing to DNA destruction, these cells were in late apoptosis stages. Necrosis was assessed by the proportion of PI-positive cells alone (Figure 4c). Similar to the cell live/dead assay discussed above, the electrotherapy group had almost no result in necrosis and apoptosis when compared with the control group. A small number of dead cells and apoptosis status cells were detected in BP groups. This might be because BP breakdown into phosphate anions in the cells, and the persistent rise of cytosolic phosphate anions affects cellular ATP hydrolysis to trigger programmed cell death. Cell necrosis and apoptosis number enlarged after laser treatment, suggesting that BP-based nanomaterials could cause A549 cell apoptosis with 635 nm laser irradiation. The statistical analysis revealed that BP L&E group exerted a more effective phototherapeutic effect than the BP L group.

**Figure 5:**
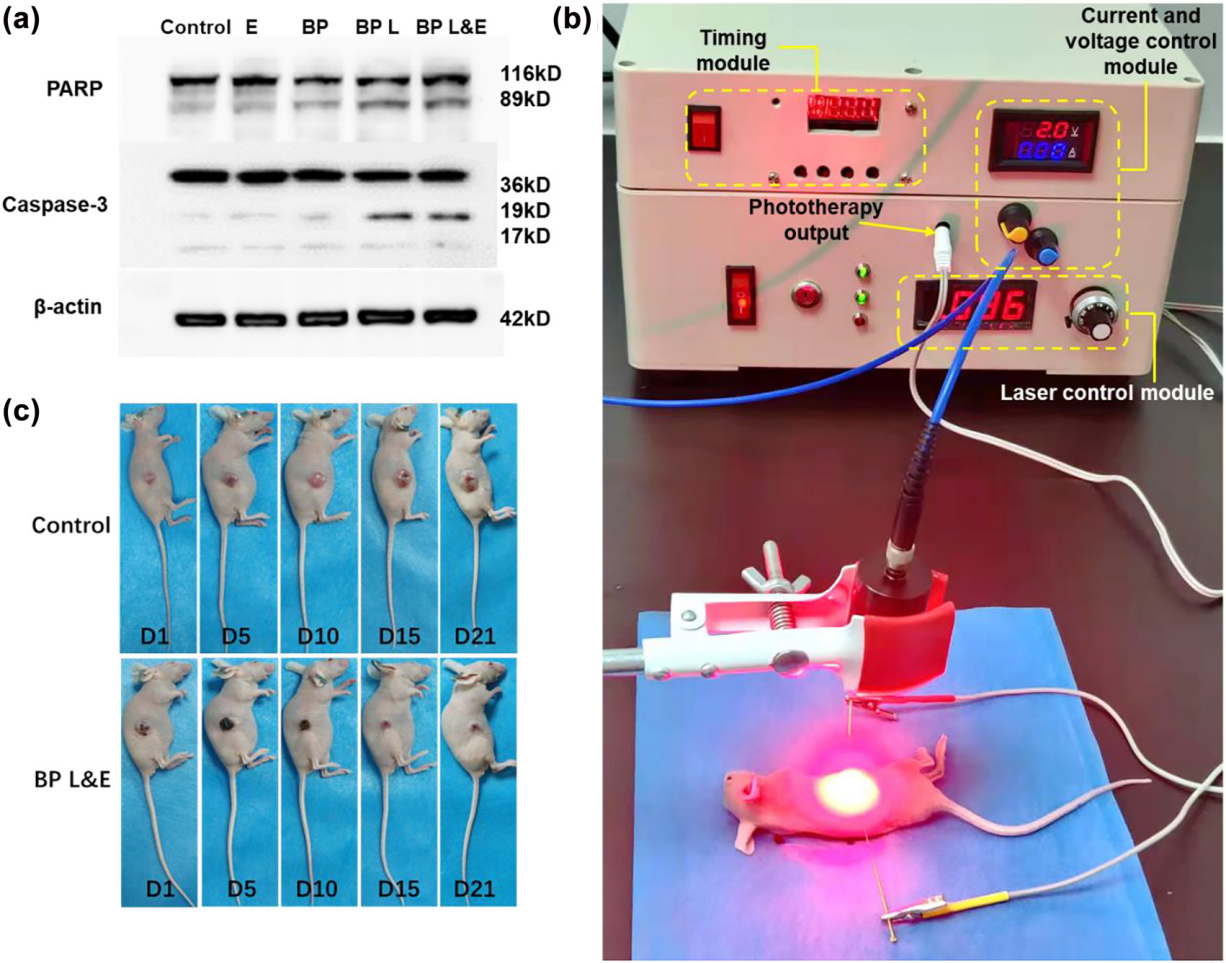
WB and in vitro analysis of BP-based nanomaterials with different treatments. (a) Western blot analysis for PARP, Caspase-3, (b) photography of self-designed multimodal cancer treatment instrument for antitumor therapy, (c) photography of tumor-bearing mice variation with time.

To evaluate the apoptosis pathway in A549 treated with BP nanomaterials, the critical apoptotic machineries, such as PARP and caspase-3 were detected using western blot. In accordance with the results of western blot (Figure 5a), cleaved caspase-3 and PARP significantly increased in BP L&E group. The WB results present the same situation as the flow cytometric results discussed previously. Among those groups, the largest amount of ROS was generated in BP L&E groups, which leads to severe cell apoptosis.

In summary, *in vitro* experiment results demonstrated that electrotherapy did enhance the production of ROS in photodynamic therapy, resulting in more dramatic apoptosis of cells.

### 3.3 In vivo experiment for A549

According to the BP-based nanomaterials presented a good anticancer performance *in vitro*, Balb/c nude mice bearing A549 tumors were established to determine the effectiveness of the anticancer of this cancer therapy further. A multimodal cancer treatment instrument was built up for this *in vivo* experiment. As shown in Figure 5b, this device mainly contains three parts, the laser control module, the electrical treatment control module, and the timing module. The 635 nm laser output power was adjusted by switching the knob on the right. The higher the displayed current, the higher the output power. There are two patterns for the electrical treatment control module: constant voltage (0.3–5 V) and constant current (0.01–1 A). The yellow and blue knobs control the current and voltage, respectively. In this paper, only a constant voltage pattern was utilized. The application duration of phototherapy and electrotherapy are regulated by the timing module.

**Figure 6:**
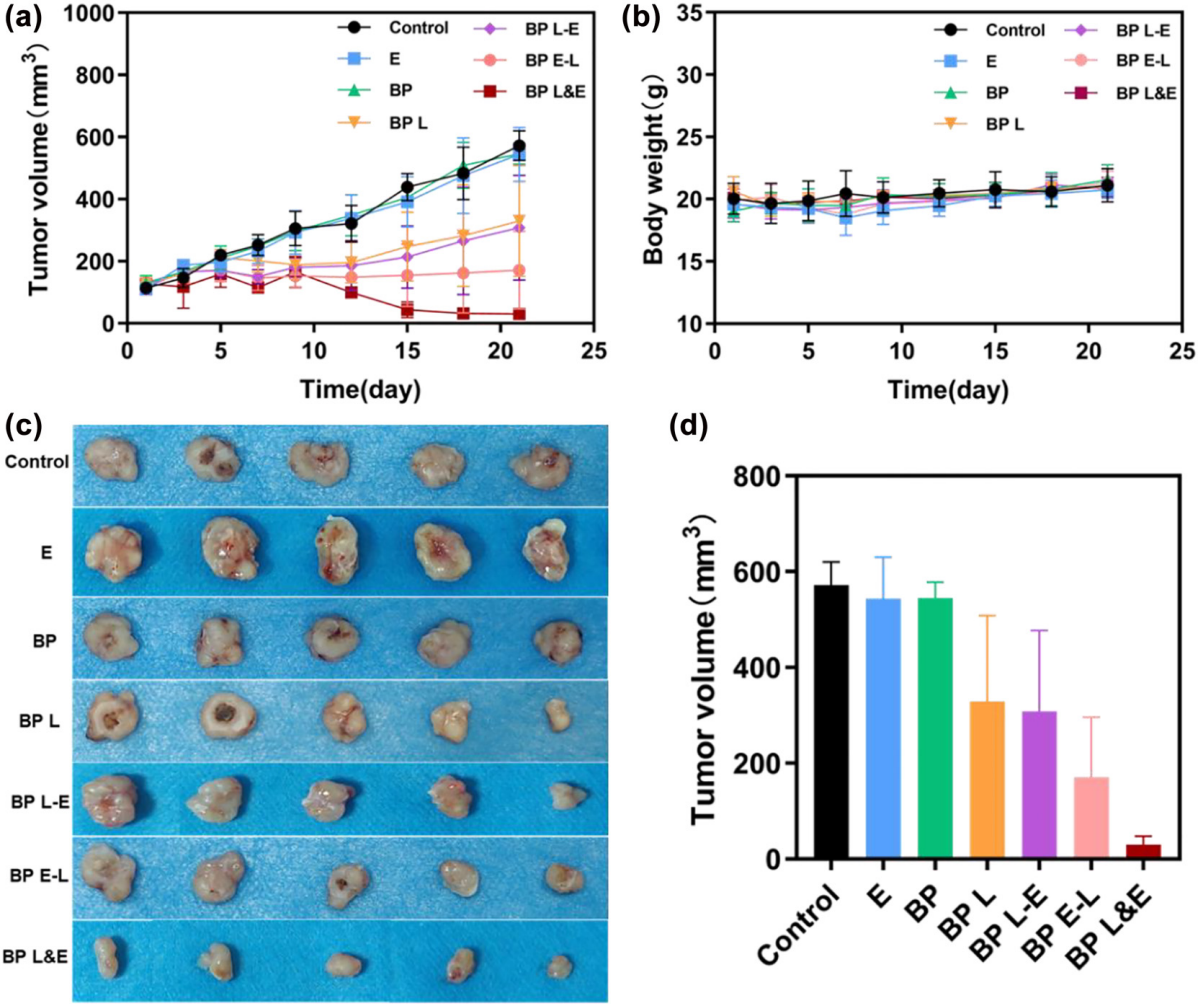
In vitro evaluations of BP-based nanomaterials post various treatments. (a) Tumor volume development over time after different treatments, *n* = 5, (b) body weight changes over time of different groups, *n* = 5, (c) images of harvested tumor tissue after 21 days, (d) statistical analysis of tumor volume on 21 days.

The treatment system was divided into seven groups and the changes in tumor volume and body weight of mice were measured over 21 days. The typical comparison between the control group and BP L&E group was shown in Figure 5c. The control group’s tumor size continuously increased during the observation period, while the BP L&E group’s tumor volume gradually decreased after treatment. The variations in tumor volume and mice body weight were presented in Figure 6a and b, respectively. Besides, the statistical analysis of tumor volume after 21 days was shown in Figure 6d. Obviously, there was no apparent difference among the control group, electrotherapy group, and BP group according to the tumor volume. For all phototherapy groups, the tumor volume growth was significantly inhibited. Tumor proliferation was inhibited to a similar extent for BP L group and BP L-E group. The possible explanation was the oxygen generated by post-electrotherapy could not participate in the ROS generation. Therefore, this further proved that the synergistic effect of electrotherapy is mainly oxygen provider. The assistance of PTT and *in situ* hydrogen generation of electrotherapy were additional benefits. The antitumor effect of BP E-L group was better than BP L group, as the part of produced oxygen interrupted ROS generation, but was limited by the complexity of oxygen concentration maintenance in TME. The BP L&E group demonstrated a distinguished antitumor effect, consistent with the conclusion of the *in vitro* studies. As in the previous study, the generated oxygen enhanced the PDT efficiency, resulting in better therapeutic results [24].

When concerning the variation within the group (Figure 6c), the BP L&E group presented the best among all groups, which suggested the good stability of this synergetic therapy. Furthermore, there was no detectable variation in the mice’s body weight among all seven groups during the entire observation period, indicating negligible side effects on the mice. In summary, this synergetic therapy exhibited remarkable antitumor effects and biocompatibility *in vivo*.

**Figure 7:**
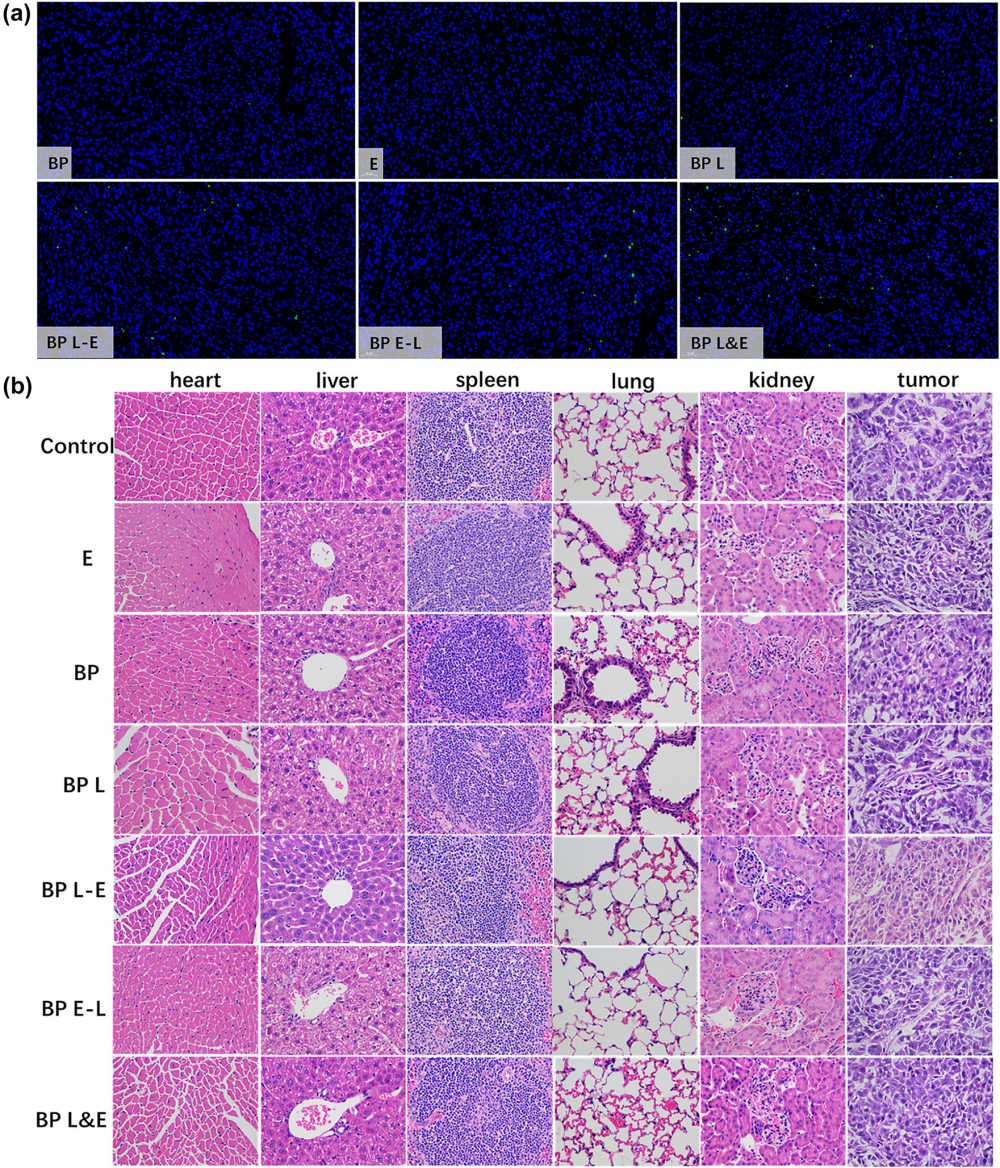
Immunohistochemistry and H&E analysis of all groups. (a) TUNEL analysis for tumor tissue sections of treated groups at 21 days, (b) H&E stained histological images of major organs and tumor tissue sections after 21 days.

The cell necrosis and apoptosis in the tumor tissues following various therapies were examined via H&E staining and the TUNEL assay (Figure 7). In Figure 7a, no TUNEL-positive tumor cell was detected in BP group and E group. This suggested BP nanomaterials and electrotherapy alone would not lead to tumor necrosis. TUNEL-positive tumor cells appeared after PDT, indicating that PDT resulting the tumor cell be killed via necrosis and apoptosis. The BP L&E group had the most severe apoptosis based on TUNEL analysis. The listed H&E staining results in Figure 7b demonstrated that neither BP nor electrotherapy induce a substantial infection or inflammation. On the basis of H&E histological images, no visible damage was observed in sections of major organs, confirming that BP-based nanomaterials are biocompatible with electrotherapy and phototherapy.

## 4 Conclusions

In this study, BP-PEG nanomaterials were successfully fabricated and used in E-PDT for anticancer purposes. Hypoxia is a clinically relevant issue in cancer therapy and oxygen is consumed continuously in PDT, which aggravates hypoxia at the tumor site. Electrotherapy, a direct and efficient method of oxygen generation via water splitting, was selected in conjunction with phototherapy. The safety of oxygen generation is much improved by avoiding additional drug intake, which might result in systemic toxic effects and finally cause side effects when collaborating with other cancer therapy. *In vivo* and *in vitro* results manifested that BP-based nanomaterials could effectively produce ROS under laser irradiation. Meanwhile, the ROS generation further increased in the presence of electrolytic oxygen production. The E-PDT with BP-based nanomaterials displayed excellent photodynamic therapeutic efficiency under 635 nm laser irradiation, achieving apoptosis of A549 cells effectively. In conclusion, this work offers a novel and secure strategy for utilizing BP-based nanomaterials in E-PDT. It demonstrates a strong potential for future clinical translation to overcome the limitations of hypoxic tumor tissue in cancer treatment.

## Supplementary Material

Supplementary Material Details

## References

[1] Monro S., Colon K. L., Yin H. (2019). Transition metal complexes and photodynamic therapy from a tumor-centered approach: challenges, opportunities, and highlights from the development of TLD1433. Chem. Rev..

[2] Pham T. C., Nguyen V. N., Choi Y., Lee S., Yoon J. (2021). Recent strategies to develop innovative photosensitizers for enhanced photodynamic therapy. Chem. Rev..

[3] Kwiatkowski S., Knap B., Przystupski D. (2018). Photodynamic therapy – mechanisms, photosensitizers and combinations. Biomed. Pharmacother..

[4] Allison R. R., Moghissi K. (2013). Photodynamic therapy (PDT): PDT mechanisms. Clin. Endosc..

[5] Yaraki M. T., Liu B., Tan Y. N. (2022). Emerging strategies in enhancing singlet oxygen generation of nano-photosensitizers toward advanced phototherapy. Nanomicro Lett..

[6] Chen D., Xu Q., Wang W. (2021). Type I photosensitizers revitalizing photodynamic oncotherapy. Small.

[7] Huang L., Zhao S., Wu J. (2021). Photodynamic therapy for hypoxic tumors: advances and perspectives. Coordin. Chem. Rev..

[8] Patel A., Sant S. (2016). Hypoxic tumor microenvironment: opportunities to develop targeted therapies. Biotechnol. Adv..

[9] Deng X., Shao Z., Zhao Y. (2021). Solutions to the drawbacks of photothermal and photodynamic cancer therapy. Adv. Sci..

[10] Chen P., Cheen J., Jheng Y. (2022). Clinical applications and consideration of interventions of electrotherapy for orthopedic and neurological rehabilitation. J. Chin. Med. Assoc..

[11] Chen C., Bai X., Ding Y., Lee I. S. (2019). Electrical stimulation as a novel tool for regulating cell behavior in tissue engineering. Biomater. Res.

[12] Jin H., Guo J., Liu J. (2017). Anti-inflammatory effects and mechanisms of vagal nerve stimulation combined with electroacupuncture in a rodent model of TNBS-induced colitis. Am. J. Physiol. Gastrointest. Liver Physiol..

[13] Genovese M. C., Gaylis N. B., Sikes D. (2020). Safety and efficacy of neurostimulation with a miniaturised vagus nerve stimulation device in patients with multidrug-refractory rheumatoid arthritis: a two-stage multicentre, randomised pilot study. Lancet. Rheumatol.

[14] Qi G., Wang B., Song X., Li H., Jin Y. (2020). A green, efficient and precise hydrogen therapy of cancer based on *in vivo* electrochemistry. Natl. Sci. Rev..

[15] Cui X., Zhao L., Yu J. (2020). Water-splitting based and related therapeutic effects: evolving concepts, progress, and perspectives. Small.

[16] Wang H., Yang X., Shao W. (2015). Ultrathin black phosphorus nanosheets for efficient singlet oxygen generation. J. Am. Chem. Soc..

[17] Qin L., Jiang S., He H., Ling G., Zhang P. (2020). Functional black phosphorus nanosheets for cancer therapy. J. Control. Release..

[18] Zhang T., Wan Y., Xie H. (2018). Degradation chemistry and stabilization of exfoliated few-layer black phosphorus in water. J. Am. Chem. Soc..

[19] Anju S., Ashtami J., Mohanan P. V. (2019). Black phosphorus, a prospective graphene substitute for biomedical applications. Mater. Sci. Eng C..

[20] Qiu M., Wang D., Liang W. (2018). Novel concept of the smart NIR-light–controlled drug release of black phosphorus nanostructure for cancer therapy. PNAS.

[21] Los M., Mozoluk M., Ferrari D. (2002). Activation and caspase-mediated inhibition of PARP: a molecular switch between fibroblast necrosis and apoptosis in death receptor signaling. Mol. Biol. Cell.

[22] Porter A. G., Jänicke R. U. (1999). Emerging roles of caspase-3 in apoptosis. Cell Death Differ..

[23] Chen Y., Lin S., Chang J. (2006). In vitro and in vivo studies of a novel potential anticancer agent of isochaihulactone on human lung cancer A549 cells. Biochem. Pharmacol..

[24] Dang S., Mo Y., Zeng J. (2022). Three birds with one stone: oxygen self-supply engineering palladium nanocluster/titanium carbide hybrid for single-NIR laser-triggered synergistic photodynamic-photothermal therapy. Nanophotonics.

