# Supplementary material for: Oxygen-supplied electrotherapy for enhanced photodynamic synergistic therapy overcomes hypoxia tumor microenvironment
Source: Nanophotonics. 2022 Oct 12;11(22):5077–88. doi: 10.1515/nanoph-2022-0417 (PMC11501713; doi:10.1515/nanoph-2022-0417)
Supplement: Supplementary file 1 — Supplementary Material Details [file j_nanoph-2022-0417_suppl.docx]

**Oxygen-supplied electrotherapy for enhanced photodynamic synergistic therapy overcomes hypoxia tumor microenvironment**

Chaozhou Li,^1^ Hui Tan,^2^* Ruitao Lu,^3^ Sainan Qin,^2^ Xiangying Meng,^3^ Han Zhang,^1^ Zhongjian Xie^2^*

Figure S1. Particle analysis of BPNSs and BP-PEG using DLS

The average particle size of BPNSs measured by DLS was ~167nm slightly larger than the results obtained using TEM and AFM analysis. The BP-PEG average particle size was ~216nm. The size distribution results for both materials were presented in Fig S2.


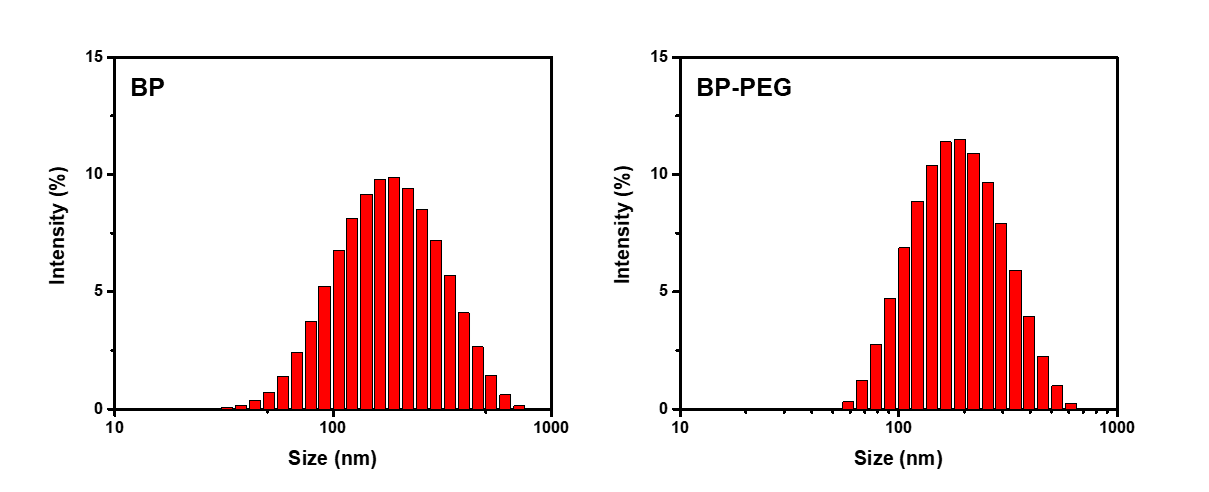


Figure S2. DLS measurements (size distribution by intensity) for both BP and BP-PEG


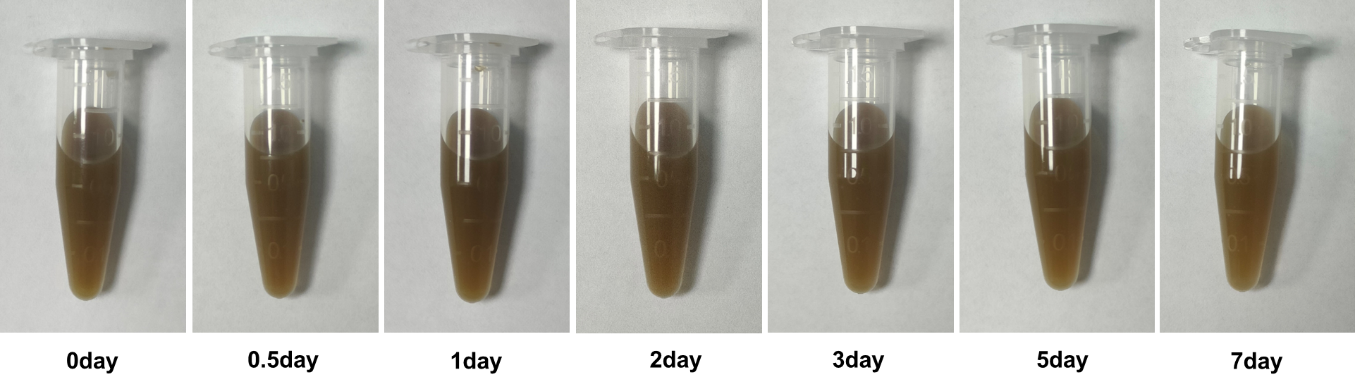


Figure S3 The stability analysis of BP-PEG suspension after electrotherapy

The prepared BP-PEG was dispersed in PBS and treated with 2V DC power for 2 minutes. Fig S3 displayed the degradation of produced BP-based nanomaterials with time, which indicated that this nanomaterial could last for 7 days post phototherapy.


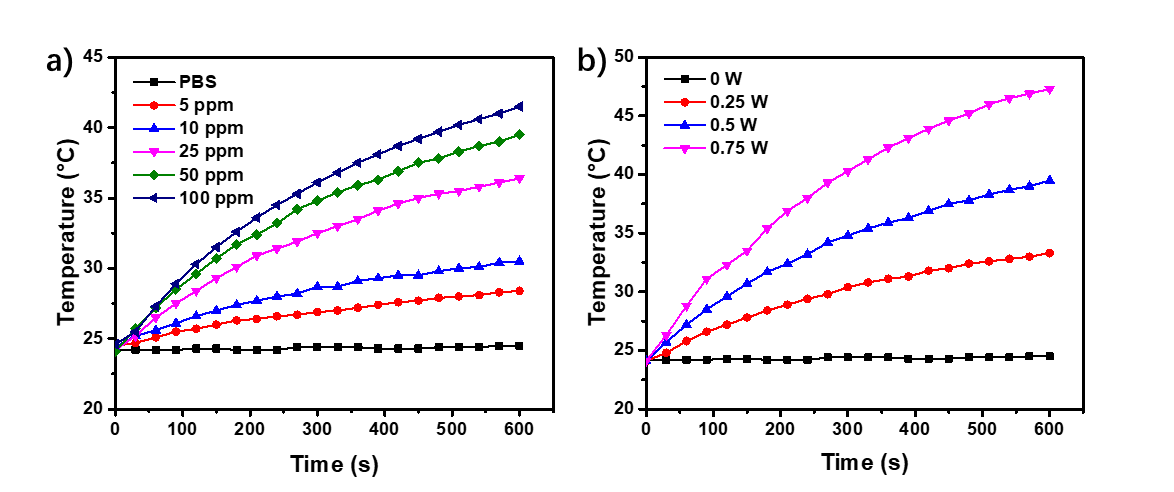


Figure S4. Photothermal profile of BP-PEG under irradiation a) with different BP-PEG concentrations with 0.5 W laser irradiation, b) with different laser intensities with 50ug/ml BP-PEG


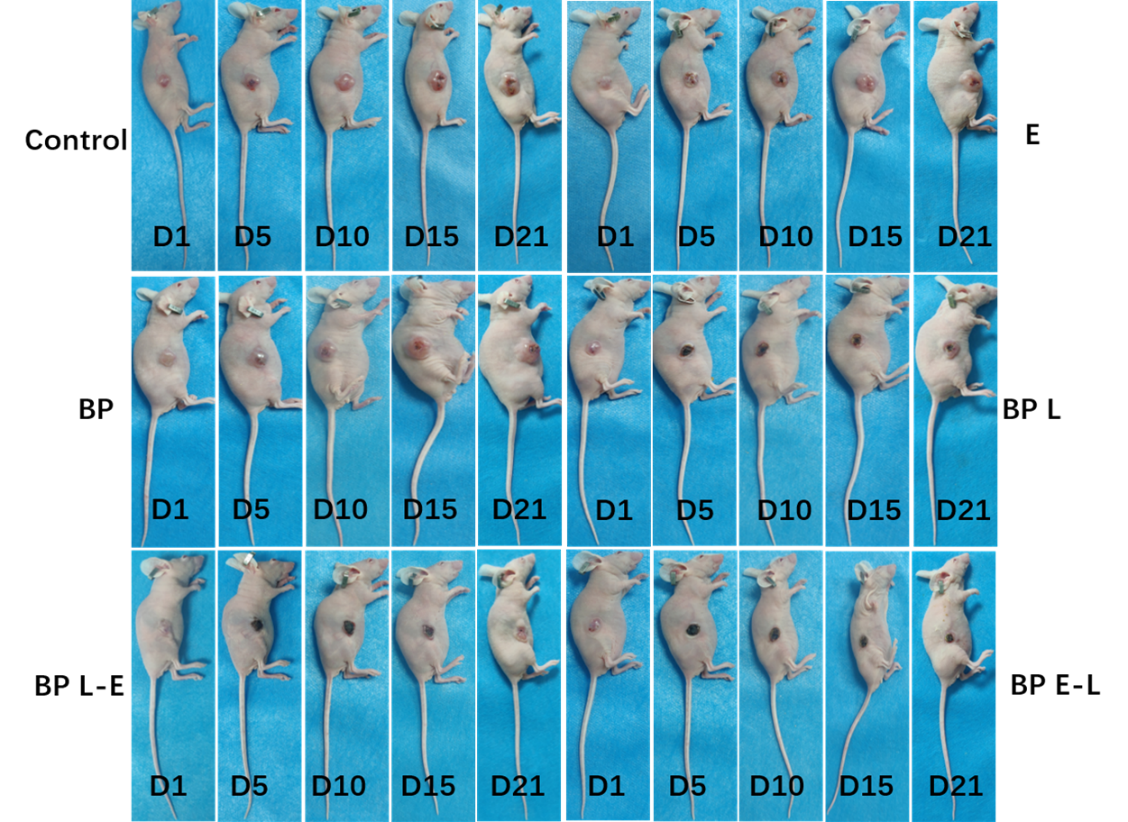


Figure S5. Photography of tumor-bearing mice variation with time after different treatments
